# The likelihood of khat chewing serving as a neglected and reverse ‘gateway’ to tobacco use among UK adult male khat chewers: a cross sectional study

**DOI:** 10.1186/1471-2458-14-448

**Published:** 2014-05-13

**Authors:** Saba Kassim, Nikki Rogers, Kelly Leach

**Affiliations:** 1 Institute of Dentistry, Queen Mary, University of London, Barts and The London School of Medicine and Dentistry, 4 Newark Street, London E1 2AT, UK; 2 Wright State University Boonshoft School of Medicine, Dayton 45420, OH, USA

## Abstract

**Background:**

Chewing khat leaves is often accompanied by tobacco use. We assessed aspects of tobacco use and explored factors associated with tobacco use patterns (frequency of use per week) among khat chewers who used tobacco only when chewing khat (“simultaneous tobacco and khat users”, STKU).

**Methods:**

A sample of 204 male khat chewers was recruited during random visits to khat outlets. Data collected included socio-demographic items, tobacco use and khat chewing behaviours. Both psychological and physical dependence on khat were assessed using the Severity of Psychological Dependence on Khat (SDS-Khat) Scale, the Diagnostic Statistical Manual IV (DSM-IV) and adapted items from the Fagerström Test for Nicotine Dependence (chewing even when ill, and difficulty in abstaining from khat chewing for an entire week). Descriptive statistics and non-parametric analyses were conducted.

**Results:**

Of the 204 khat chewers, 35% were khat chewers only, 20% were STKU, and the remainder were daily cigarette smokers. The mean age of STKU was 38.12 (±14.05) years. Fifty seven percent of STKU smoked tobacco and chewed khat for two days per week and 43% smoked and chewed more frequently (three to six days: 33%, daily: 10%). Three quarters (74%) were former daily tobacco users. Khat chewing initiated tobacco smoking among 45% of STKU and 71% reported attempts to quit tobacco smoking during khat chew. Among STKU, smoking tobacco for more than two days per week was significantly associated (p < 0.05) with psychological dependence (increased levels of SDS-Khat), physical dependence (increased levels of DSM-IV symptoms, chewing even when ill, difficulty in abstaining from chewing for an entire week and self-reported health conditions) and behavioural factors (e.g. amount of khat chewed in typical khat session).

**Conclusions:**

Khat chewing may promote different patterns of tobacco smoking, initiate and sustain tobacco smoking, and trigger tobacco cessation relapses among STKU. Increased frequency of tobacco smoking among STKU was linked to psycho-physical and behavioural factors. Further investigation within large and representative samples of both sexes of STKU in different contexts should be considered for health research and policy development. Khat chewing should be considered when designing tobacco prevention uptake, cessation interventions and relapse prevention programmes.

## Background

The World Health Organization has recommended that pro-tobacco social influences should be addressed [1] and that more countries need to take the necessary steps to reduce tobacco use to save the lives of the billion people who may otherwise die this century from tobacco-related illnesses worldwide [2]. Factors reported to contribute to tobacco uptake, either separately or as an adjunct to other substances, include socio-economic factors, ethnicity/culture and shared genetic and environmental factors [3-5].

Chewing khat leaves, which have ‘amphetamine like’ effects [6], is a social activity practiced by Yemenis, Somalis, Ethiopians and other East African communities both in their original countries and within the diasporas [7,8]. Khat chewing sessions are often held in groups that segregate male and female chewers based on cultural norms [9]. These sessions, which are often held in the afternoon and may continue for 5–6 hours, play socio-cultural roles that include resolving social conflicts and exchanging ideas [10]. Other functions of khat chewing include enhancing concentration among students when studying [11] and, among females, the relieving of social and mental stress [12] and demonstration of the passage into womanhood.

khat chewing has recently become a debated issue among politicians and public health policy makers within individual countries and internationally [13]. While khat chewing is associated with socio-economic factors (e.g. family budget constraints) [14] and environmental impacts (replacement of essential crops with khat and depletion of water resources for khat agriculture) [15,16], it is also associated with a number of unfavourable health outcomes including cardiovascular disease [9]. One of the most common consequences of khat chewing is tobacco uptake [17,18].

In general, current evidence for the health impacts of khat chewing combined with tobacco use is scarce, and the available evidence presents conflicting conclusions. Al-Motarreb and colleagues’ case–control study [19] reported that khat chewing and tobacco smoking were independent risk factors for myocardial infarction (MI). Khat chewing has also been reported, as an independent risk factor for oral mucosa changes (i.e. white lesions), irrespective of tobacco use [20,21]. Importantly, the evidence for the combined impacts of khat and tobacco amongst khat chewers who use tobacco only when chewing khat is currently unavailable. Nevertheless, tobacco use is an established common risk factor for many non-communicable diseases [22].

Tobacco use among khat chewers in various populations is reported as regular or only during khat chewing [17,18,23-26]. The estimated prevalence of tobacco use among khat chewers which covers the time frame of use (e.g. last three or six months) and a clear definition (i.e. daily versus other use) is currently under review, as is the estimated prevalence of tobacco use among khat chewers who only use tobacco when chewing [27,28].

Although khat chewers who only smoke whilst chewing may be labelled “episodic smokers”, [25], we named this group “simultaneous tobacco and khat users” (hereafter STKU) to better reflect the reporting of khat and tobacco use methods and the context and time of tobacco use only when using khat. Tobacco can be used in different forms (e.g. cigarette, waterpipe [shisha] and smokeless tobacco). Similarly, khat is most often chewed but can be used as tea, paste, paste with honey, occasionally smoked, taken as capsules [29-31] or even drunk as a juice.

Some studies indicate that khat chewers increase their tobacco intake both overall and during khat chewing sessions [8,17,25], while others observed a compulsive need to smoke among naïve khat chewers who socialised with habitual smoker chewers and that current daily smokers may have initiated tobacco smoking due to khat chewing [32,33]. High percentages of both daily cigarette smoker khat chewers and STKU self-reported that smoking tobacco whilst chewing was important for enhancing the impacts of khat [25,34]. Randomised control trials assessing this in different contexts of khat use are not currently available.

A knowledge gap exists concerning tobacco use among STKU. In particular, there is a dearth of information regarding aspects of tobacco use that includes initiator data and patterns of tobacco use (frequency of tobacco). Additionally, factors associated with different patterns of tobacco use among STKU have not been explored.

Neglecting tobacco use embedded within khat chewing may lead to damaging tobacco-related effects of this behaviour going unnoticed in these populations. This is especially important for khat chewers who use tobacco only when chewing khat. The custom of using tobacco during khat chewing can jeopardise abstinence among former tobacco users, increase likelihood of tobacco initiation amongst non-users and contribute to unsuccessful attempts to quit tobacco use when chewing khat. Understanding the patterns of simultaneous tobacco and khat use, especially within the STKU sub-population, will aid our ability to measure public health impacts and develop effective prevention and cessation programs. Our aims in this study were 1) to assess aspects of tobacco use among STKU that included pattern of use (frequency of use per week), initiators and history of use (former daily tobacco use) and attempts to quit tobacco whilst chewing and 2) to explore the underlying factors associated with patterns of tobacco use among STKU. We hypothesized that socio-demographic, psycho-physical and behavioural factors may lead to different patterns of tobacco use among STKU.

## Methods

Study methods and sampling were adopted to explore several health outcomes including the aspects of tobacco use among STKU. The methods are described fully in our previous work [10,25,35] and summarised below.

### Study design and sample selection

This was a cross sectional study that recruited a purposive sample of 204 male khat chewers from outlets of khat sale in the UK. Female khat users were not recruited because cultural norms keep a majority of them from public association with khat. Random sampling of the Yemeni community is reported as difficult (Sheffield Hallam University, unpublished observation). As such we recruited our sample via random visits to khat sale outlets. This strategy was recommended by Taylor and Griffiths [36], where, in defining a drug use population, their physical and geographical sample point at a pre-specified time may if necessary be used instead of having a prior exhaustive sampling frame. The selection criteria for recruitment into the study were Yemeni individuals aged 18 years or older who spoke Arabic or English, chewed khat at least once a week over the preceding 12 months, voluntarily provided a sample of carbon monoxide (CO) for analysis, had no health conditions that deterred their participation and had permanent residency in the UK.

We recruited only male Yemeni chewers as there was a dearth of information on khat chewing and tobacco use in this community compared to other chewing communities (e.g. the Somali community) [37]. We excluded temporary residents (visitors and students on visa entry) because the study sought to extrapolate the findings of this study to other U.K. resident Yemeni khat chewers [25]. Chewers who did not meet the other inclusion criteria above were excluded from the study.

### Data collection instruments

Data were collected via structured, validated face-to-face interviews [35] that investigated the socio-demographics, khat chewing behaviours, khat dependence [10,38,39] and aspects of tobacco use among STKU. Both the Diagnostic Statistical Manual IV (DSM-IV) [38] and Severity of Psychological Dependence on Khat (SDS-Khat) [10,39] scales were used to assess chewers’ khat dependence. The SDS-khat consists of five items with responses measured on a Likert-type scale, and scores from 0–3 with a possible total score of 15. It focuses on impaired control over, and preoccupation and anxiety about drug use [39]. We derived seven questions from the Structure Clinical Interview for the DSM-IV modified for khat chewing to assess aspects of physical and behavioural dependence. These included the domain of tolerance and withdrawal symptoms, substance taken in large amounts, and unsuccessful attempts to cut out or reduce the amount of khat use. A full description of these questions is reported elsewhere [10]. Scores of three and more were considered as demonstrating DSM-IV symptoms.

Additionally, two items that reflect physical dependence were modified from the Fagerström Test for Nicotine Dependence [40]. These included the inability to go one whole week without khat chewing. The time frame of one week (rather than one day) was used to accommodate chewers who chew more frequently as well as those who chewed less frequently (e.g. once a week). We also used the item “chewing even when ill”. Khat is thought to be less potent but not dissimilar to substances like tobacco [41] with respect to its psychoactive properties [6] and socio-behavioural reinforcement [42]. Khat is also perceived as a remedy for many symptoms including asthma, depression and social isolation [37]. Chewers were also asked to self-report health condition (s).

With respect to tobacco use [35] an opening question, ‘Are you currently a regular (daily) tobacco user?’ was asked. Chewers who replied ‘No’ were asked a follow up question, ‘Do you currently use tobacco during khat chewing?’. Chewers who responded positively were then asked other questions that included: ‘Can I just check which of the following you currently consider your primary tobacco product?’; ‘Have you ever been a regular user of the following tobacco products {e.g. cigarettes)?’; ‘How many cigarettes/cigars do you currently smoke in a khat session?’; ‘If you smoke waterpipe [shisha], do you smoke your own shisha?’; ‘Do you share waterpipe [shisha] with others?’; ‘Do you smoke more cigarettes/cigars or waterpipe [shisha] during the first hours of a khat session rather than during the rest of the session?’; ‘Who of the following initiated your smoking cigarettes/cigars or waterpipe [shisha] use?’; and finally, ‘Have you ever tried to give up cigarettes/cigars or waterpipe [shisha] as a khat chewer?’. Self-reported tobacco use status was validated with CO recordings.

### Ethical approval

The study was reviewed and approved by the East London and City Health Authority Local Research Ethics Committee. Participants were individually briefed about the study and provided with an information sheet explaining the aims and methods of the study. Participants signed an informed consent form prior to the interview, and information obtained was coded anonymously to optimise data confidentiality.

### Data analysis

The Statistical Package of the Social Sciences (SPSS) version 20 was used to analyse the data. Descriptive statistics were calculated to report sample characteristics. Patterns of tobacco smoking were categorised into two groups: Group 1 = one to two days per week tobacco smoking only when chewing khat, and Group 2 = three and more days per week tobacco smoking only when chewing khat. This categorisation accommodates for the small number of daily smoker chewers in this sample and is based on the report that chewing for more than two days per week is problematic [43].

Bivariate analysis using non-parametric chi square or Fisher exact tests (employed for cells with counts less than 5) were used to compare proportions for categorical data in relationship with patterns of tobacco use among STKU and to obtain the odds ratios with a 95% confidence interval (95% CI). Mann–Whitney U tests were used to compare the medians of continuous variables across categorical variable (patterns of tobacco us among STKU) when the continuous variable data was not normally distributed. The level of significance (p-value) was two tailed and set at p <0.05.

## Results

### Baseline sample characteristics

We screened 219 khat chewers of whom 204 met the criteria for inclusion. We only encountered Yemeni khat chewers with the exception of one Somali male who was excluded from the study. Among the 204 recruited male khat chewers, 20% (n = 42) were STKU, 45% were daily cigarette smokers and the remainder were khat chewers who did not use tobacco. Table 1 shows the baseline characteristics of the whole sample with regard to tobacco use status. As for the characteristics of the STKU, as the main focus of this study, the mean age of STKU was 38.12 (±14.05) years. Of the STKU, 57% (n = 24) smoked and chewed khat for two days a week, 33% (n = 14) smoked and chewed between 3 and 6 days a week and the remainder were daily smoker chewers. Sixty percent (N = 25) reported smoking cigarettes as their primary tobacco, 7% smoked both cigarette and waterpipe and 33% smoked only waterpipe. In addition, 74% (n = 31) were former daily tobacco users (cigarettes, waterpipe or traditional waterpipe [Mada’a] and smokeless tobacco [shamma]). Finally, 45% of the STKU reported that khat chewing initiated their tobacco smoking, 41% (n = 17) smoked more tobacco during the first hours of chewing versus the later hours and 33% (n = 14) continued tobacco smoking after expectorating the khat bolus.

**Table 1:** Baseline characteristics of khat chewers per tobacco use status (n = 204)

**Variable**	**STKU**^**d **^**(n = 42) N (%) or Mean ± SD**	**Daily cigarette smoker chewers (n = 91) N (%) or Mean ± SD**^**a**^	**Khat chewers only (n = 71) N (%) or Mean ± SD**
**Age**	38.12 ± 14.05	40.36 ± 17.84	53.55 (21.85)
**Marital status**			
Married	34 (81.0)	62 (68.1)	61 (85.9)
In other status	8 (19.0)	29 (31.9)	10 (14.1)
**Employment**			
Employed	19 (45)	32 (35.2)	21 (26.9)
Unemployed	23 (55)	59 (64.8)	50 (70.4)
**Education**			
High level	20 (48)	32 (35.2)	18 (25.4)
Low level	22 (52)	59 (64.8)	53 (74.6)
**Social participation**	42 ± 18.90	40.00 ± 21	42.34 ± 17.93
**Daily time of chewing khat**			
1-3 PM	19 (45.2)	37 (40.7)	36 (50.7)
After 3 PM	23 (54.8)	54 (59.3)	35 (49.3)
**Former daily tobacco use**			
No	11 (26)	------------------	------------------
Yes	31 (74)		
**Initiator of tobacco smoking**			
Social (e.g. friends)	23 (54.76)	81 (89.01)	------------------
Environmental (khat setting)	19 (45.24)	10 (10.99)	
**Ever tried quitting khat chewing**			
No	19 (45.2)	47 (51.6)	30 (42.3)
Yes	23 (54.8)	44 (48.4)	41 (57.7)
**Ever tried quitting TS**^**b **^**when chewing**			
No	12 (28.6)	------------------	------------------
Yes	30 (71.4)		
**Age starting khat chewing**	19.50 ± 5.48	18 ± 6.00	19.36 ± 7.11
**Days chewing per week**	2.67 ± 1.79	3.30 ± 2.08	2.72 ± 1.90
**Hours chewing per session**	6.08 ± 1.95	6.00 ± 2.09	5.52 ± 1.81
**Khat amount per session in bundle**	1.85 ± 1.05	1.51 ± 0.50	1.37 ± 0.50
**Cigarettes smoked per session**	15.07 ± 10.33^e^	21.04 ± 13.36	-----------------
**Own waterpipe smoked per session**	2.89 ± 1.96	-----------------------	----------------
**Attempts to quit TS**^**b **^**when chewing**	2.77 ± 1.94	-----------------------	----------------
**Attempts to quit khat chewing**	3.13 ± 2.07	4.20 ± 4.44	3.63 ± 2.92
**CO PPM scores**	16 ± 15.66	20.53 ± 12.12	-----------------
**SDS-khat scores**	5.36 ± 4.38	5.08 ± 3.57	6.16 ± 4.35
**DSM-IV scores**	1.42 ± 1.87	1.60 ± 1.84	1.92 ± 2.10
**Chewing even ill**			
No	29 (69.0)	50 (54.9)	46 (64.8)
Yes	13 (31.0)	41 (45.1)	25 (35.2)
**Smoke more in the first hours of chewing**			
No	25 (59.5)	55 (60.4)	----------------
Yes	17 (40.5)	36 (39.6)	
**Chew more in the first two hours**			
No	31 (73.8)	65 (71.4)	31 (43.7)
Yes	11 (26.2)	26 (28.6)	40 (56.3)
**Easy/difficult not chewing the whole week**			
Easy	30 (71.4)	53 (58.2)	37 (52.1)
Difficult	12 (28.6)	38 (41.8)	34 (47.9)
**Want to quit chewing**			
No	19 (45.2)	47 (51.6)	44 (62.0)
Yes	23 (54.8)	44 (48. 4)	27 (38.0)
**Health conditions**^**c**^			
No	30 (71.4)	62 (68.1)	35 (49.3)
Yes	12 (28.6)	29 (31.9)	36 (50.7)

^a^SD, Standard deviation; ^b^TS, tobacco smoking; ^c^health conditions = heart problems, diabetic and asthma; ^d^STKU = simultaneous tobacco and khat users; ^e^a combination of cigarette and waterpipe tobacco smoker (WPTS) chewers considered for 1 participant from Group1 and 2 participants from Group 2, mean/SD of only cigarette smoked when excluded cigarette and WPTS chewers was 15.84±10.54.

As for khat chewing behaviour variables among STKU the mean age of khat chewing initiation was 19.50 ± 5.48 years old, the mean/±SD for DSM-IV dependence on khat chewing was 1.42 ± 1.87 (median = 1, range 1–7) and 19% (n = 9) met the criteria for DSM-IV khat dependence (scores 3 and more). Thirty-one percent (n = 13) of the STKU chewed khat even when ill, 26% (n = 11) chewed more khat in the first two hours, 29% (n = 12) would find it difficult to spend the whole week without chewing khat and 55% (n = 23) reported wanting to quit chewing khat. Table 1 shows other aspects of khat chewing and tobacco use among STKU.

### Factors associated with patterns of tobacco use among STKU

Table 2 shows a greater number of days of simultaneous tobacco smoking and chewing per week (Group 2) was significantly associated with a greater amount of khat chewed per session and increase in khat dependence levels measured by DSM-IV and SDS-Khat (p = 0.001, p = 0.015, p = 0.002, respectively).

**Table 2:** **Mann Whitney U test results of factors associated with pattern of tobacco smoking among STKU**^**d**^

**Variable**	**N**	^**e**^**M ± SD, Mdn**	**Mean rank**	**Sum of ranks**	**P-value**	**Effect size**
**SDS-khat**						
^a^Group 1	24	3.25 ± 3.67, 2.00	15.90	381.50	0.001	0.53
^b^Group 2	18	8.17 ± 3.68, 8.00	28.97	521.50		
**DSM-IV**						
Group 1	24	0.83 ± 1.37,0.00	17.77	426.50	0.015	0.35
Group 2	18	2.22 ± 2.18, 2.00	26.47	476.50		
^**c**^**Amount of khat chewed during typical khat session**						
Group 1	24	1.44 ± 0.90, 1.00	16.56	397.50	0.002	0.48
Group 2	18	2.39 ± 1.01, 2.50	28.08	505.50		
^**c**^**Number of cigarettes smoked when chewing**						
Group 1	15	13.00 ± 5.59,14.00	14.20	213.00	0.856^e^	0.032
Group 2	13	17.46 ± 13.85,10.00	14.85	193.00		
^**c**^**Khat chewing session hours last**						
Group 1	24	6.30 ± 1.66,6.00	23.19	556.50	0.270	0.17
Group 2	18	5.78 ± 2.29,6.00	19.25	346.50		
**Age starting chewing khat**						
Group 1	24	20.00 ± 5.87,20.00	23.25	558.00	0.282	0.17
Group 2	18	18.06 ± 4.86,19.00	19.17	345.00		

^a^Group 1 = 1-2 days per week tobacco smoking when only chewing khat; ^b^Group 2 = 3 and more days per week tobacco smoking when only chewing khat; ^c^Time frame last 12 months; ^d^STKU = simultaneous tobacco and khat users; ^e^M ± SD, Mdn = mean/standard deviation, median; ^e^a combination of cigarette and waterpipe tobacco smoker chewers considered for 1 participant from Group1 and 2 participants from Group 2, no differences observed in number of cigarette smoked amongst both groups when excluded WPTS and cigarette smoker chewers (p= 0.467).

Chi square and Fisher’s exact tests (Table 3) show that STKU who smoked three or more days per week during khat sessions (Group 2) when compared with those who smoked one or two days per week during khat sessions (Group 1) were more likely to consume more khat in the first two hours of khat session (44.4% versus 12.5%, OR = 5.60, 95% CI = 1.22, 25.75, p = 0.033), chew khat when ill (55.6% versus 12.5%, OR = 8.75, 95% CI = 1.90,40.24, p = 0.006), find it very difficult to spend a whole week without chewing (55.6% versus 12.5%, OR = 13.75, 95% CI = 2.46, 76.82, p = 0.006), report wanting to quit chewing (72.2% versus 41.7%, OR = 3.64, 95% CI = 1.00, 13.52, p = 0.049) and to report health conditions (44.4% versus 16.7, OR = 4.00, 95% CI = 1.00, 16.55, p = 0.049). Neither other socio-demographic (e.g. employment status) and behavioural variables, nor previous attempts to quit tobacco smoking when chewing khat were significantly associated (p > 0.05) with patterns of tobacco smoking among STKU.

**Table 3:** **Chi square and fisher exact test results of observed differences in pattern of tobacco use among STKU**^**d**^

**Variable**	^**a**^**Group 1 N = 24 N (%)**	^**b**^**Group 2 N = 18 N (%)**	**OR (95% CI)**	**P-value**
^**c**^**Chewing more khat during the first two hours of khat session**				
Yes	3 (12.5)	8 (44.4)	5.60 (1.22, 25.75)	0.033
^**c**^**Chewing even ill**				
Yes	3 (12.5)	10 (55.6)	8.75 (1.90,40.24)	0.006
^**c**^**Want to quit chewing**				
Yes	10 (41.7)	13 (72.2)	3.64 (1.00, 13.52)	0.049
^**c**^**Attempted to quit chewing**				
Yes	11 (45.8)	8 (44.4)	0.95 (0.28,3.23)	0.929
^**c**^**Whole week not chewing**				
Difficult	2 (7.3)	10 (55.6)	13.75 (2.46,76.82)	0.001
**Health conditions**				
Yes	4 (16.7)	8 (44.4)	4.00 (1.00,16.55)	0.049

^a^Group 1 = 1-2 days per week tobacco smoking when only chewing khat; ^b^Group 2 = 3 and more days per week tobacco smoking when only chewing khat; ^c^Time frame last 12 months; ^d^STKU = simultaneous tobacco and khat users.

## Discussion

This is the first study to investigate aspects of tobacco use in STKU that included initiation and patterns of tobacco use, former use of tobacco and attempts to quit tobacco when chewing khat. The likelihood of khat chewing among STKU acts as a ‘gateway’ and relapse trigger to tobacco smoking was highlighted for the first time. Psycho-physical and behavioural factors associated significantly with patterns of tobacco use among STKU.

### Confirmation and bridging knowledge gaps of the current literature

The overall consensus based on emerging evidence is that khat may be a ‘gateway drug’ to tobacco [44]. This sample of STKU lends further support to the theory that khat chewing may be a ‘gateway’ to tobacco smoking through smoking triggers activated by chewing among a number of chewers [17,18,24]. Further analysis of daily smoker chewers (n = 91) within this whole study sample shows that the mean age of initiation of khat chewing is younger than that of tobacco use initiation (18 for khat chewing versus 19 years for tobacco smoking). Although 28% initiated tobacco at the same age as they began khat chewing, 37% reported that their tobacco use began after the initiation of khat chewing. Even accounting for recall bias, longitudinal prospective studies should be considered in future research to explore the role [25] of khat chewing as a causal of tobacco use among khat chewers of both sexes in different contexts.

This study also highlights for the first time that khat chewing can also promote different patterns of tobacco use among this subsample (STKU). A significant percentage of the identified STKU in this study reported chewing and smoking tobacco more than two days a week and the participants reported that khat chewing can be a trigger for tobacco smoking cessation relapse. A significant number of chewers in this sample had once been former daily tobacco users who reported return to tobacco use when in the environment of khat chewing. This finding is crucial particularly for designing tobacco cessation programmes for Yemenis and populations of East African descendants as khat chewing is embedded in different aspects of their socio-cultural life. We hypothesise that tobacco use among khat chewers follows a cyclical pattern: although current daily tobacco users initiated tobacco when chewing khat [25,33], our findings suggest a large proportion of STKU were former daily tobacco users. The study did not however specify a time frame for relapse.

Additionally, it is established that attempts to quit tobacco smoking are a proxy for motivation to stop smoking [45]. A significant number of STKU reported unsuccessful attempts to quit smoking tobacco when chewing khat. One should take into account the role of environment characteristics in facilitating tobacco use as well as hindering successful tobacco cessation [5,46]. Khat chewing is a socially rooted behaviour amongst Yemenis and other East African populations [42]. Khat is often chewed collectively and in places where tobacco is often used [17,18]. Importantly, smoking tobacco is perceived among khat chewers as enhancing khat impacts, a fact which should be considered as another barrier to quitting tobacco among both STKU and daily smoker chewers [25,34].

With respect to factors associated with patterns of tobacco use among STKU, our earlier report [10,25] found that increased khat dependence levels (psychological or physical) were associated with increased nicotine dependence levels among daily cigarette smoker chewers. This study explored these observations only within the context of STKU, who also demonstrated increased levels of khat dependence (as measured by SDS-Khat and DSM-IV) with more frequent khat chewing and tobacco smoking (Table 2). Although assessment of tobacco dependence was not undertaken among these STKU, aspects of dependence include the frequent use of a dependence creating substance (i.e. tobacco) [47]. Assessment of tobacco dependence among STKU should consider the cross-cultural validity of available measures or, alternatively, consider substance-specific dependence assessment [48,49].

It is important to note that the interrelationship between khat chewing and tobacco use is still under-researched. A recent work on the neurobiology of concomitant khat and tobacco use [50] suggests that both nicotine and cathinone (the active chemical in khat) act upon the reward pathway in the mesolimbic area of the brain, increasing dopamine release from the nucleus accumbens [51-56]. As such chewing khat (e.g. Mirra or Harari khat) with different levels of components (e.g. higer or lower levels of psychotropic Cathedulins) may differentially impact on the reward pathway and Cathinone [57]. This may not only trigger tobacco use but also increase the potential for different nicotine dependence levels among khat chewers [50]. This is suggested by the fact that the reported number of cigarettes smoked among cigarette smoker chewers was higher than among smokers only [21] and number of cigarettes smoked among chewers who smoked when chewing only were less than among daily smoker chewers [25]. Levels of tobacco dependence among smoker chewers of both sexes using different brand of khat is unexplored and likewise the shift to high nicotine yield products separately (e.g. cigarettes) or combined (e.g. cigarettes and waterpipe) and its impacts on tobacco cessation among tobacco khat users.

Greater amounts of khat chewed weekly were associated with patterns of tobacco use among STKU. It is well established that tolerance (an increase in the amount of a drug necessary to achieve the desired effects), is one aspect of drug dependence [58]. Moreover, the association of frequent tobacco use and khat chewing among STKU with increased amount of khat chewed at the beginning of a khat session may imply a potential overlap of both khat and tobacco withdrawal symptoms that this group may encounter. While an understanding of such behaviour has not been explored before, we may make inferences from the current literature on drug dependence. Daily cigarette smokers were more likely to smoke within the first hour after waking, and to smoke more after waking up to restore nicotine levels [40].

Moreover, the inability to abstain from khat chewing for a whole week, the practice of chewing when even ill and the desire to quit chewing were all explored for the first time as factors associated with patterns of tobacco use among STKU. It is formally and informally reported that abstinence from khat chewing is associated with a range of withdrawal symptoms [10]. As such, these observed behaviours may reflect the accentuated impact of combined tobacco use and khat chewing that sustains both frequent tobacco use and khat chewing among STKU. Finally, the association of health conditions with patterns of tobacco use among STKU is highlighted in this study. Due consideration should be given to the social dimensions of khat and associated tobacco use [17], the belief that chewing khat is beneficial in alleviating certain health conditions [35,37] and the provision of symptomatic medical care to khat chewing communities [37,59]. The overlooked health implications khat-associated tobacco use by CTKU and the potential of this pattern as a gateway to tobacco dependence should be more thoroughly explored.

### Limitations of the study and future research directions

There were limitations to this study. First, the under-powered sample size meant that type II errors may have occurred and rendered association between potential factors (e.g. socio-economic status) and our outcome of interest (patterns of tobacco use among STKU insignificant). That is, a lack of power may have lead us to accept the null hypothesis of no significant association between socio-economic status and tobacco and khat use patterns. Likewise, the inflated percentages reported among this STKU should be interpreted cautiously. The cross-sectional nature of the study limited us to exploring association rather than causality [60]. Female chewers were excluded from the study because female chewers are less likely to be found in khat outlets due to cultural taboo. It is possible that khat chewers who have legally immigrated might have different khat chewing and tobacco use patterns than those who are undocumented or who are not yet legal citizens.

This study highlighted a number of knowledge gaps. It is well known that daily tobacco smokers will often smoke their first cigarette of the day during within hours of waking [40]. However, STKC delay tobacco intake until starting khat chewing. Long lasting effects of khat [61] may compensate for the withdrawal symptoms of nicotine, specifically among frequent STKU. Randomised control trials may elucidate such observations and may shed light on whether khat chewing or tobacco use is the primary drug of dependence among STKU. A high percentage of self-reported daily khat chewers admitted to hospital for acute myocardial infarction were shown to have the urge/desire to smoke [62]. We may also hypothesize that the combination effects of both khat and tobacco to sustain withdrawal symptoms may be an alternative explanation. Additionally, the number of cigarettes smoked among STKU, sleeping pattern[63], number of hours chewing khat [25,26,33] and continuing to smoke after expectoration of khat bolus [25] and other psychosocial factors may play a role in delaying the uptake of the first cigarette/or other form of tobacco among STKU who smoked frequently (more than two days).

The delay in tobacco intake among STKU could also be a result of classical conditioning [50]: increased activity in the hippocampus and amygdala while smoking tobacco creates the mental association between khat chewing and smoking tobacco [56]. As such, the classical model of early intake of tobacco to restore the level of nicotine [40] may not be applicable to STKU. Current observations suggest that waterpipe and Latino tobacco smokers are less likely to smoke daily and after waking [64-67]. These aforementioned hypotheses with respect to time of tobacco intake among STKU should be investigated separately or as adjuncts to other factors to develop further our understanding of tobacco use and dependence in culturally diverse populations including this subsample of khat chewers. Finally, there is a lack of information on aspects of tobacco use among pregnant women and female khat chewers as well as on hospital patients (diagnosed or under-diagnosed with mental health) and students who use tobacco only when chewing khat. The history of tobacco use among khat chewers who do not use tobacco currently should be investigated thoroughly in future research. In this study groups both the SDS-khat and DSM-IV scores (Table 1) among older participants who only chewed khat was higher than other groups.

### Policy implications

The National Institute for Health Care and Excellence (NICE) guidelines for the UK recommend that tobacco intervention should focus on the needs of minorities and incorporate culturally appropriate interventions [68]. These preliminary findings may guide and inform effective health care prevention and promotion efforts among East African and Arab immigrants and refugees who now make their homes in the UK. Healthcare professionals involved with these communities should be aware of such patterns of tobacco use among STKU who may not consider their tobacco use great enough to report. As such, an opportunity for providing the tobacco cessation advice recommended in the UK Quality and Outcomes Framework [69] may therefore be missed.

To enhance behavioural support changes in smoking cessation programmes, it may be prudent to screen for khat chewing in Yemeni, Somali and other khat chewing ethnic groups considering the role khat may play in smoking relapse. In other words, to maintain sustainable tobacco abstinence, behavioural support changes among this group should be combined with enhancement of self-efficacy and not ‘a puff rules’ [70,71]. Khat chewers are exposed to cues for tobacco use embedded in khat chewing, whether living in the diasporas or when going for occasional visits to their homelands. This also has global health implications, especially for countries like Yemen, Ethiopia and Somalia, where both tobacco smoking and khat chewing are endemic and tobacco control efforts are not well established [72].

## Conclusions

Khat chewing may promote different patterns of tobacco smoking, initiate and sustain tobacco smoking, and trigger tobacco cessation relapses among STKU. Increased weekly frequency of tobacco smoking among STKU was linked to psycho-physical and behavioural factors. Further investigation of these findings within large and representative samples of both sexes of STKU in different contexts should be considered for health research and policy development. Future research is required to address identified knowledge gaps with respect to patterns of tobacco use among STKU.

## Abbreviations

STKU: Simultaneous tobacco and khat users; CO: Carbon monoxide; SDS-khat: The severity of psychological dependence on khat scale; DSM-IV: The diagnostic statistical manual IV.

## Competing interests

All authors declare that they have no conflicts of interest. This study was supported by the U.K. Ambassador of the United Arab Emirates Sheik Issa Al-Kark, Princess Fadaw Bent Khalid, and Dr. Elham Danish Head of the Cultural Department in Saudi Arabia Embassy, U.K. These parties have no role in the study design, data collection, analysis or interpreting, writing of the manuscript or the decision to submit the manuscript for publication.

## Authors’ contributions

SK was the principal investigator, reviewing the literature, collecting, analysing the data, interpreting the results, drafting the article and addressed reviewers’ questions. Both NR and KL assisted in literature review and draft writing. All authors contributed to manuscript revisions and approved the final draft.

## Pre-publication history

The pre-publication history for this paper can be accessed here:

http://www.biomedcentral.com/1471-2458/14/448/prepub
